# Translated Mutant *DSPP* mRNA Expression Level Impacts the Severity of Dentin Defects

**DOI:** 10.3390/jpm12061002

**Published:** 2022-06-19

**Authors:** Youn Jung Kim, Yejin Lee, Hong Zhang, Figen Seymen, Mine Koruyucu, Sule Bayrak, Nuray Tuloglu, James P. Simmer, Jan C.-C. Hu, Jung-Wook Kim

**Affiliations:** 1 Department of Pediatric Dentistry & DRI, School of Dentistry, Seoul National University, Seoul 03080, Korea; ykim71@snu.ac.kr (Y.J.K.); lyj72255621@gmail.com (Y.L.); 2 Department of Biologic and Materials Sciences & Prosthodontics, School of Dentistry, University of Michigan, Ann Arbor, MI 48109, USA; zhanghon@umich.edu (H.Z.); jsimmer@umich.edu (J.P.S.); janhu@umich.edu (J.C.-C.H.); 3 Department of Paediatric Dentistry, Faculty of Dentistry, Altinbas University, Istanbul 34147, Turkey; figen.seymen@altinbas.edu.tr; 4 Department of Pedodontics, Faculty of Dentistry, Istanbul University, Istanbul 34116, Turkey; mine.yildirim@istanbul.edu.tr; 5 Private Practice, Eskisehir 26150, Turkey; suleb76@yahoo.com (S.B.); nuraytuloglu@yahoo.com (N.T.); 6 Department of Molecular Genetics & DRI, School of Dentistry, Seoul National University, Seoul 03080, Korea

**Keywords:** hereditary, splicing mutation, dentinogenesis imperfecta, dentin sialophosphoprotein, *DSPP*, silent mutation, genotype−phenotype relationship

## Abstract

Hereditary dentin defects are conventionally classified into three types of dentinogenesis imperfecta (DGI) and two types of dentin dysplasia (DD). Mutations in the dentin sialophosphoprotein (*DSPP*) gene have been identified to cause DGI type II and III and DD type II; therefore, these are not three different conditions, but rather allelic disorders. In this study, we recruited three families with varying clinical phenotypes from DGI-III to DD-II and performed mutational analysis by candidate gene analysis or whole-exome sequencing. Three novel mutations including a silent mutation (NM_014208.3: c.52-2del, c.135+1G>C, and c.135G>A; p.(Gln45=)) were identified, all of which affected pre-mRNA splicing. Comparison of the splicing assay results revealed that the expression level of the *DSPP* exon 3 deletion transcript correlated with the severity of the dentin defects. This study did not only expand the mutational spectrum of *DSPP* gene, but also advanced our understanding of the molecular pathogenesis impacting the severity of hereditary dentin defects.

## 1. Introduction

Tooth dentin is formed by odontoblasts, which are ectodermally-derived mesenchymal cells that differentiate under reciprocal molecular interactions with ameloblasts to produce mineralized dentin during the lifetime of the tooth [1]. Resulting from a series of ectodermal mesenchymal interactions, dentin is formed to make up the bulk of the tooth. The coronal dentin within the tooth crown is covered and protected by enamel, which is the hardest tissue in the human body mediated by the ameloblasts. Whereas the root dentin is covered by cementum, a thin, uniquely calcified tissue anchoring the tooth root to the alveolar bone via the periodontal complex. Inside the tooth resides the pulp tissue, containing nerves and blood vessels that govern the vitality and sensitivity of the tooth [2].

The mechanical properties and chemical composition of dentin are similar to those of bone; however, dentin has a unique microstructure with parallel dentinal tubules [3]. Dentinal tubules are filled with an elongated cell process of odontoblasts and dentin-forming cells and span the entire thickness of dentin from the dentino-enamel junction to the pulp chamber. During tooth development, a collagen-rich predentin matrix is secreted by odontoblasts and a confluent layer of dentin mineralizes near the opposing layer of ameloblasts (enamel-forming cells). Dentin expands on the predentin (odontoblast) side of the initial dentin mineral while secreted enamel proteins, in close proximity to the ameloblast cell membrane, initiate enamel mineral ribbon formation on the ameloblast side of the initial dentin [4]. Dentin and enamel formation are closely coordinated and specific genetic defects can be deleterious to the formation of both dentin and enamel [5,6].

Hereditary dentin defects were previously documented as dentinogenesis imperfecta presenting with various syndromic dentin defects accompanied by fragile bone diseases, osteogenesis imperfecta (OI) [7], or hereditary opalescent dentin, indicating an isolated dentin pathology without any other symptoms [8]. Currently, the most used classification system is the one proposed by Shields et al. in 1973 when they reported a new disease entity, dentin dysplasia type II [9]. 

The Shields classification system consists of two categories and five types: three types of dentinogenesis imperfectas (DGI-I, DGI-II, and DGI-III) and two types of dentin dysplasia (DD-I and DD-II) [9]. DGI-I is an OI-related disease entity. In contrast, the other types are nonsyndromic, characterized by marked discoloration and attrition in both dentitions (deciduous and permanent) and pulp chamber obliteration after tooth eruption or sometimes even before tooth eruption. Other characteristics include bulbous crowns with marked cervical constriction and increased attritions. However, not all OI patients present with DGI-I and the expressivity varies even in a patient; thus, the degree of discoloration and pulp chamber obliteration can vary among teeth. Clinical and radiographic findings of DGI-II have many similarities to DGI-I, but the penetrance is almost complete and the expressivity is much more consistent. This is one of the common dental genetic diseases that affects approximately one in every 8000. DGI-III is for clinical characteristics found in the Brandywine triracial isolate from southern Maryland and Washington DC. They present additional features such as multiple pulp exposures in deciduous teeth and widely varying forms of pulp chambers from total obliteration to normal to even a shell teeth-like appearance. DD-I is somewhat unique compared to the other types in terms of the discoloration, shape and pulp chamber obliteration characteristics. In most cases, teeth are clinically normal in color and shape in both deciduous and permanent dentition. However, radiographically, the teeth exhibit short roots with crescent-shaped remaining pulp chambers parallel to the cemento-enamel junction, and multiple periapical radiolucencies can be seen in non-carious teeth. Histologically, the pulp chamber is filled by a characteristic abnormal dentin with the appearance of “lava flow around the boulder” [10]. DD-II shares characteristic features with DGI-II in deciduous dentition; however, the permanent dentition is normal in shape and discoloration is minimal, if any. Although, radiographically, the pulp chamber of the permanent dentition shows obliteration with characteristic thistle-tube-shaped remnants and/or pulp stones.

The Shields classification system was constructed based on clinical and radiologic findings. The types are well-defined and the characteristic features are informative of the nature of diseases for the pathologists and dental clinicians. Subsequent clinical and genetic studies expanded the understanding of hereditary diseases affecting tooth dentin. It has been known that other skeletal diseases other than OI can cause defective dentin like DGI-I [11,12,13]. Mutations in the *DSPP* gene have been identified in many cases of DGI-II, DGI-III, and DD-II [14,15,16,17,18]. It has been shown that DGI-III is not just for the Brandywine triracial isolate and DGI-III features (multiple pulp exposure and a shell teeth-like appearance) are not unique to this specific population [19,20,21]. Similar phenotypes caused by different *DSPP* mutations and varying but overlapping phenotypes with the same *DSPP* mutation suggest genetic and/or nongenetic modifiers that warrant further molecular genetic analysis [22,23].

In this study, we recruited three families with different clinical phenotypes of hereditary dentin defects and performed mutational analysis by candidate gene analysis or whole-exome sequencing. The objective was to determine and compare the effect(s) of the *DSPP* mutations to advance our understanding of the molecular pathogenesis of hereditary dentin defects.

## 2. Materials and Methods

### 2.1. Human Subject Enrollment and Genomic DNA Isolation

The institutional review boards of Seoul National University Dental Hospital (CRI05003G and 9 December 2021), Istanbul University (No: 2008/931 and 20 September 2019), and the University of Michigan (H03-00001835-M1 and 6 May 2021) independently reviewed and approved this study. The informed consent was obtained from all participating individuals after explaining the nature of the study. Pedigrees were drawn according to the family histories taken. Clinical examination and saliva sample collection were performed according to the principles in the Declaration of Helsinki. Genomic DNA was isolated by a conventional method and the quality and quantity were measured.

### 2.2. Candidate Gene Sequencing and Whole-Exome Sequencing 

Candidate gene analysis was done for the DNA sample of the proband (II:2) of family 2, as described before [24]. Exons and exon−intron boundaries of the *DSPP* gene were amplified by PCR, and Sanger sequencing was performed to identify any possible disease-causing variants.

DNA samples of the proband (II:1) of family 1 and the affected mother (I:2) and a sibling (II:2) of the proband of family 3 were submitted for whole-exome sequencing to BGI (Family 1; Shenzhen, China) and Johns Hopkins University Center for Inherited Disease Research (Family 3; CIDR, Baltimore, Maryland, USA). After exome capturing, paired-end sequencing reads were generated.

### 2.3. Bioinformatic Analysis

Obtained sequencing reads were processed using a series of bioinformatic analyses, as previously described [25] (Table S1). Briefly, the reads were trimmed to remove the adapter sequences and aligned to the reference human genome assembly (hg38). Bioinformatics analysis programs, such as Samtools and Genome Analysis Tool Kit, were used to get a list of sequence variants [26,27]. Annotation of the sequence variants were performed against the dbSNP build 147 database and a criterion for the minor allele frequency (MAF) of 0.01 was applied to filter the annotated variants. The identified mutations and the segregation among the family members were confirmed by Sanger sequencing. Sanger sequencing was performed for all participating family members at Macrogen (Seoul, Korea) or Eurofins Genomics (Louisville, KY, USA). The identified mutations were submitted to the ClinVar database (https://www.ncbi.nlm.nih.gov/clinvar/ (accessed on 18 May 2022), Accession ID: SCV002104181, SCV002104182 and SCV002104183).

### 2.4. Mutagenesis and In Vitro Splicing Assay

A wild-type *DSPP* genomic fragment that included exons 2, 3, and 4 cloned into the pSPL3 splicing vector [28] was mutated by PCR mutagenesis to introduce the mutations identified in the study families (Table S2) and the sequence was confirmed by direct sequencing. COS-7 cells were transiently transfected with the wild-type and mutant pSPL3 vectors and total RNA was isolated after 36 h. RT-PCR (sense 5′-TAGAGTCGACCCAGCACCAT-3′ and antisense 5′-CCTCGTTTCTACAGGAATTCTCA-3′) was performed using EZ PCR master mix (Elpis, Daejeon, Korea). Amplification bands were excised from an agarose gel following electrophoresis, purified, and characterized by direct DNA sequencing. The experiments were repeated twice and the band intensities were measured twice using ImageJ (https://imagej.nih.gov/ij/ (accessed on 18 May 2022)). One-way ANOVA was used to determine whether there were statistically significant differences between the means of the wild-type and mutant splicing groups and Tukey HSD test was used for the post-hoc tests (http://vassarstats.net/ (accessed on 18 May 2022)).

## 3. Results

### 3.1. Clinical Phenotype and Diagnosis of Family 1

**Figure 1:**
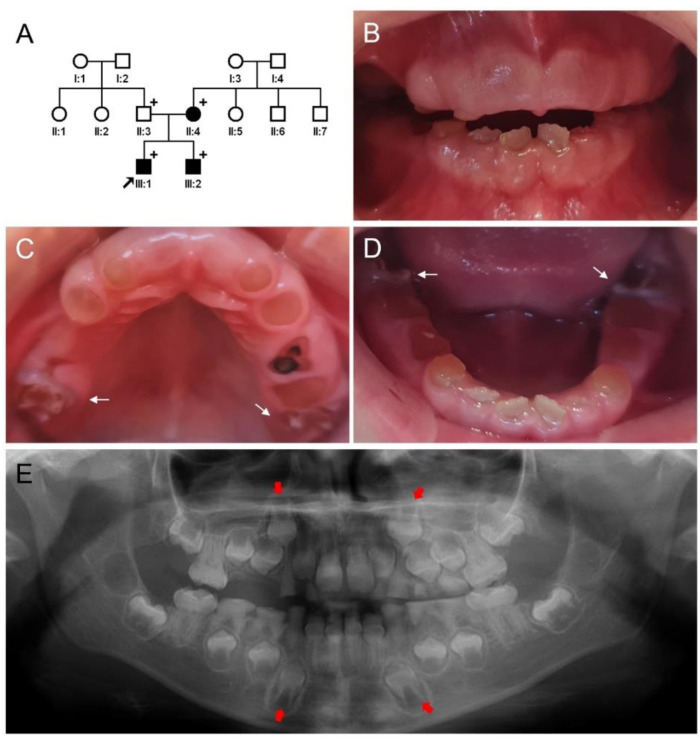
Pedigree, clinical photos, and panoramic radiograph of family 1. (**A**) Pedigree of family 1. Black symbols indicate affected individuals and the proband is indicated by a black arrow. Plus signs above the symbols indicate participating individuals. (**B**–**D**) Clinical photos of the proband (III:1) at age 8. Weak deciduous dentition shows severe attrition in most teeth to the level of gingiva. His erupting permanent mandibular anterior teeth exhibit amber−brown discoloration. The first molars (white arrows) show defective enamel formation. (**E**) Panoramic radiograph of the proband at age 8 shows severe attrition and complete pulp obliteration in all deciduous teeth. Enamel defects in the developing dentition, especially in the maxillary and mandibular canines (red arrows), can be seen radiographically.

The proband of family 1 was a 5-year-old male from a nonconsanguineous marriage of Turkish families (Figure 1). His dentition presented amber−brown discoloration with generalized severe attrition and an anterior open bite. However, there were no multiple pulp exposures or wide pulp chambers (Figure S1). At age 8, the deciduous dentition showed severe attrition in most teeth to the level of gingiva. His erupting permanent mandibular incisors exhibited amber-−brown discoloration. The first molars showed developmental enamel defects and the enamel coverage of the tooth crown was not even nor complete, exposing underlying dentin irregularly. Defective enamel coverage in the first molars could be identified in the panoramic radiograph at age 5 and the incisal area of the developing canines had an abnormal shape in the panoramic radiograph at age 8. The sibling (III:2) of the proband also showed similar clinical and radiographic findings. The mother (II:4) of the proband had full mouth prosthodontics with multiple extractions. The remaining teeth had short and blunt roots with complete obliteration. According to the family history, it could be possible that the mutation occurred spontaneously in the mother because the maternal grandparents were reportedly normal without any dental problems. There were no remarkable medical and dental histories including but not limited to bone fragility, blue sclerae, and hearing problems. Therefore, the diagnosis of DGI-II with enamel defects was assigned to family 1. 

### 3.2. Clinical Phenotype and Diagnosis of Family 2

**Figure 2:**
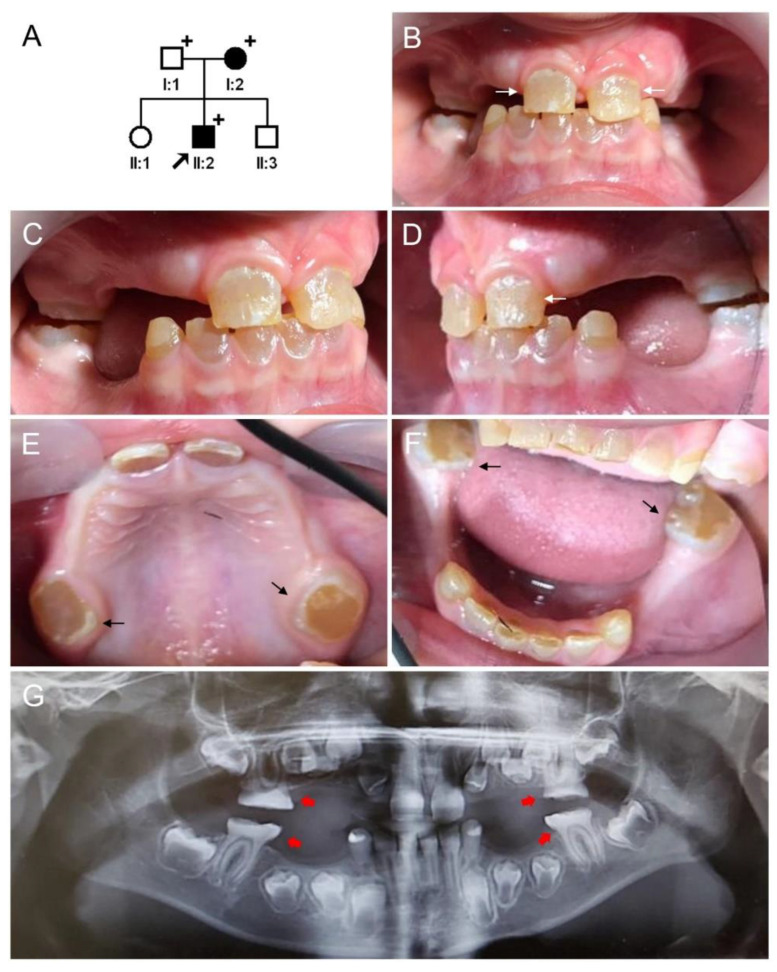
Pedigree, clinical photos, and panoramic radiograph of family 2. (**A**) Pedigree of family 2. Black-filled symbols indicate affected individuals and the proband is indicated by a black arrow. Plus signs above the symbols indicate participating individuals. (**B**–**F**) Clinical photos of the proband (II:2) at age 9. All of his deciduous teeth except the mandibular canines are missing due to severe attrition. Thin enamel (white arrows) can be seen in the maxillary central incisors and severe attrition can be seen in the first molars (black arrows). (**G**) Panoramic radiograph of the proband at age 9 shows severe attrition in first molars (red arrows). The enamel appears thin and mineralization of the enamel and dentin also appears to be reduced.

The proband of family 2 was the second child from a nonconsanguineous marriage of Turkish families (Figure 2). When he presented to the dental clinic at age 4, his discolored deciduous dentition showed generalized severe attrition to the level of gingiva except for the mandibular anterior teeth (Figure S2). Multiple pulp exposures and periapical abscesses, especially in the maxillary deciduous molars and incisors, had occurred. Abnormal enamel shape and reduced mineralization also could be identified radiographically, especially in the developing first molars. His permanent dentition at age 9 showed thin enamel in the maxillary central incisors and severe attrition of the first molars. The mother was also affected and had full mouth prosthodontics with several teeth extracted. Her remaining teeth had short and blunt roots with complete pulpal obliteration. The other two siblings of the proband had normal dentition and there were no other remarkable medical histories among all family members. The diagnosis of DGI-III with enamel defect, therefore, was established for family 2.

### 3.3. Clinical Phenotype and Diagnosis of Family 3

**Figure 3:**
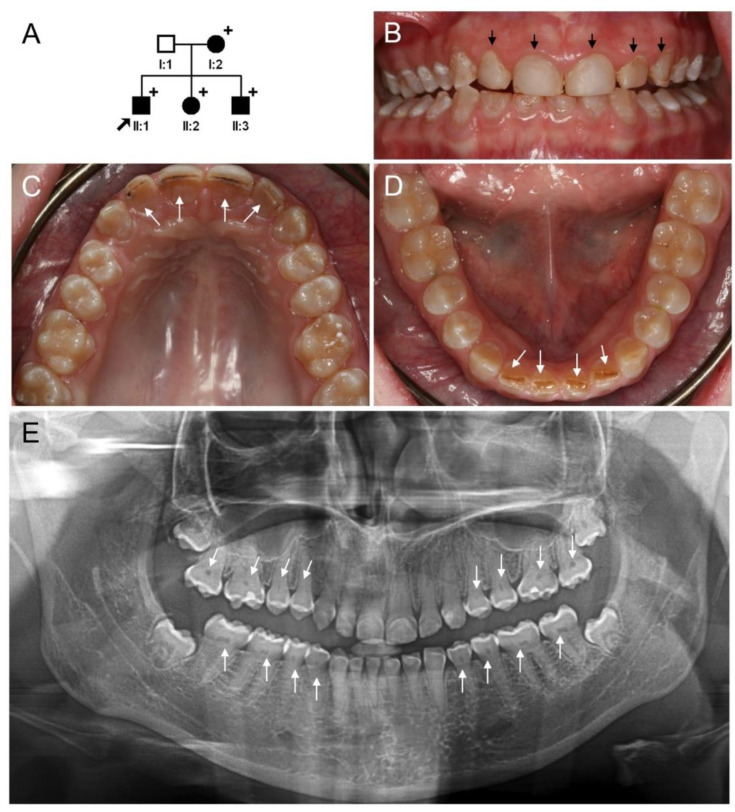
Pedigree, clinical photos, and panoramic radiograph of family 3. (**A**) Pedigree of family 3. Black symbols indicate affected individuals and the proband is indicated by a black arrow. Plus signs above the symbols indicate participating individuals. (**B**–**D**) Clinical photos of the proband (II:1) at age 18. Mild discoloration can be seen in the anterior teeth. Labial surfaces of the maxillary anterior teeth have been restored with resin composite (black arrows). Increased attrition and dentin exposure can be seen in the maxillary and mandibular incisors (white arrows). Posterior teeth are normal without discoloration and attrition. (**E**) Panoramic radiograph of the proband at age 18 years shows partial pulpal obliterations resembling thistle-tube-shaped pulp chambers (white arrows).

The proband of family 3 was an 18-year-old first-born child from a Caucasian family with no known consanguinity (Figure 3). Only mild discoloration was noticed in the anterior teeth of the proband. Labial surfaces of the maxillary anterior teeth were treated with resin composite. There were increased attritions and slight dentin exposure in the maxillary and mandibular incisors. Deep bite occlusion could be an aggravating factor. Posterior teeth were normal without discoloration or attrition. Panoramic radiograph showed partial pulpal obliterations resembling thistle-tube-shaped pulp chambers. The 17-year-old sister (II:2) of the proband also exhibited similar dental features (Figure S3). 

However, the incisor attrition was minimal and pulpal obliterations have progressed in almost all teeth compared to the proband. The 15-year-old brother (II:3) of the proband had a similar phenotype. The pulpal obliteration of his teeth was almost complete in the crown portion, but the pulp canals in the tooth roots could still be appreciated. The thistle-tube-shaped pulp chambers were not distinctive. Given the mild phenotype and characteristic pattern of pulp chamber obliteration, DD-II was designated as the diagnosis of family 3. 

### 3.4. Identification and Characterization of the Mutations of Family 1

**Figure 4:**
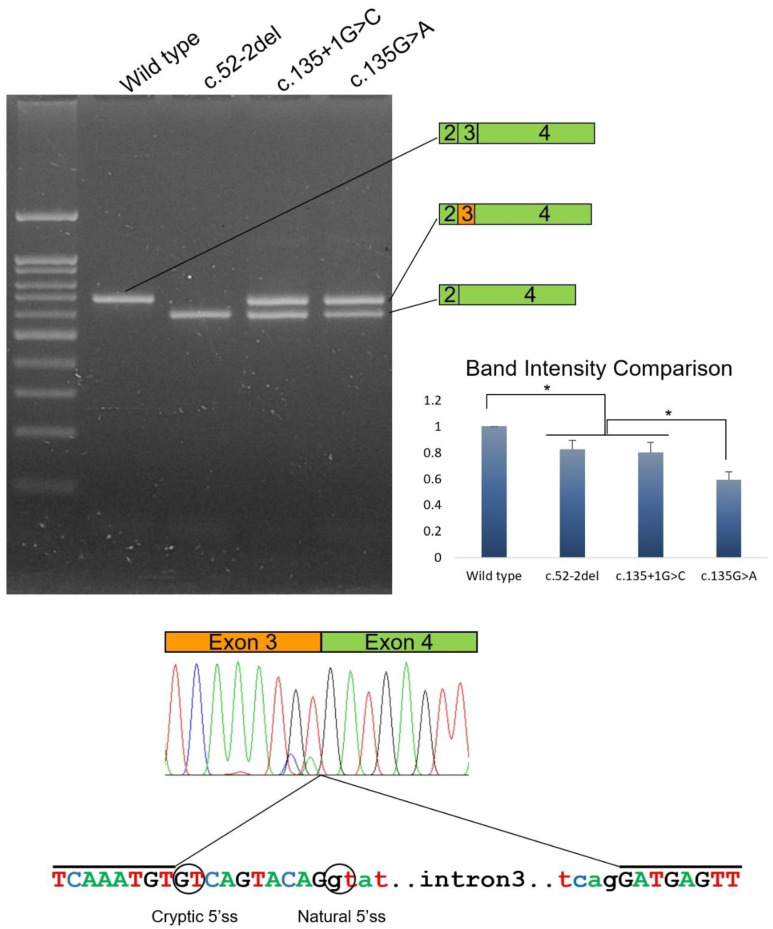
Agarose gel image of the splicing assay of the wild-type and mutants. Left lane is the DNA ladder. Wild-type and mutant names are shown above the gel image. Wild-type sequence resulted in normal splicing products including exons 2, 3, and 4. Deletion mutation (c.52-2del) resulted in an exon 3 deletion. Mutations c.135+1G>C and c.135G>A resulted in two bands: alternative splicing band using a cryptic 5′ splicing site (a shorter exon 3 with orange color) and an exon 3 deletion band. Band intensity comparison shows that the exon 3 deletion band intensity of the c.135G>A mutation is significantly weaker than the other two mutants (asterisk indicates *p* < 0.01). Intensity of the wild type band was stronger than the exon 3 deletion band of all the mutants (asterisk indicates *p* < 0.01). Sequencing chromatograms of the alternative splicing band are shown below. Abnormal, shorter exon 3 sequences are indicated with an orange box above the chromatogram. Cryptic and natural 5′ splicing sites are indicated with black circles. The sequences included in the chromatogram are indicated with black lines above the sequences.

A filtered list of the exome variant annotation of the proband of family 1 resulted in a novel deletional mutation in the *DSPP* gene but no variants in genes encoding type I collagen (*COL1A1* and *COL1A2*) [29]. The identified mutation was a single nucleotide deletion of the completely conserved splicing acceptor site (AG) at the −2 position in intron 2 (NM_014208.3: c.52-2del) of the *DSPP* gene (Figure S4). The mutation and segregation within family members were confirmed by Sanger sequencing. The result of the mutation would be a change of the splicing acceptor site from CAG to GCG. A splicing assay confirmed this deletional mutation destroyed the splicing acceptor site which resulted in the deletion of exon 3 (Figure 4). The deletion of exon 3 would result in an in-frame loss of 28 amino acids in the DSP domain (NP_055023.2: p.(Val18_Gln45del)) and the change of the conserved Ile-Pro-Val (IPV) motif to the Ile-Pro-Asp (IPD) sequence.

### 3.5. Identification and Characterization of the Mutations of Family 2

Candidate gene analysis of the *DSPP* gene identified a transversional change of guanine to cytosine at the +1 splicing donor site. Mutations to adenine or thymine at this position were previously reported [6,14,16]. This newly identified change is novel, which would destroy the completely conserved splicing donor site (TG) in intron 3 (NM_014208.3: c.135+1G>C) of the *DSPP* gene (Figure S5). The mutation and segregation were confirmed by Sanger sequencing. A splicing assay resulted in two bands with similar intensity. Use of the cryptic splicing donor site in exon 3 instead of the destroyed natural splicing donor site by the mutation resulted in a larger band (Figure 4). As a result, 10 nucleotides would be missing from the normal exon 3 sequence; therefore, it would cause a frameshift and introduce a premature stop codon in exon 4 [30]. This mutant transcript using the cryptic splicing site would be degraded due to the premature stop codon by the nonsense-mediated mRNA decay surveillance system (NMDS) [31,32]. The other smaller band was confirmed as the transcript with the exon 3 deletion, similar to that of the mutational effect identified in family 1.

### 3.6. Identification and Characterization of the Mutations of Family 3

Selection and filtering of the shared exome variants between two affected individuals resulted in a novel synonymous change in the *DSPP* gene but no variants in *COL1A1* and *COL1A2* (Figure S6). The identified variant was a transitional change of guanine to adenine at codon position 45 (NM_014208.3: c.135G>A). This change would not alter the amino acid codon at this position (NP_055023.2: p.(Gln45=)); however, it was assumed that it could affect pre-mRNA splicing because its location was at the end of exon 3. A splicing assay showed this mutation caused abnormal splicing events like the mutation in family 2. 

### 3.7. Comparison

When the mutational effect on splicing was tested, it seemed that the exon 3 deletion transcript band was much weaker than the alternative splicing band using the cryptic splicing donor site in family 3. Comparison of the splicing assay revealed that the exon 3 deletion transcript of the silent mutation in family 3 was weaker than the other two exon 3 deletion bands of the mutations in families 1 and 2 (*p* < 0.01) (Figure 4). 

## 4. Discussion

In this report, we identified three novel mutations in three families with hereditary dentin defects. Family 1 was DGI-II and the mutation was a single nucleotide deletion destroying the splicing acceptor site in intron 2 (c.52-2del) of the *DSPP* gene. The mutation resulted in an exon 3 deleted mutant transcript only. It seemed that there was no alternative splicing site in the intron 2 acceptor site; therefore, the disruption of the original splicing site resulted in an exon 3 deletion only. The in-frame deletion of 28 amino acids encoded by exon 3 in the DSP domain (NP_055023.2: p.(Val18_Gln45del)) would change the conserved IPV motif to the IPD sequence [33]. It has been demonstrated that this mutant transcript resulted in intracellular retention in the rough endoplasmic reticulum (ER) and greatly reduced secretion into culture media [16,34]. 

The mutation in family 2 was a splicing donor site mutation at the +1 position in intron 3 (c.135+1G>C) of the *DSPP* gene. Because there was a cryptic splicing donor site in exon 3 [30], this splicing mutation resulted in two transcripts. However, the mutant transcript using the cryptic splicing donor site would not produce a truncated protein because of the NMDS. Therefore, the exon 3 deleted mutant transcript would only be translated like the mutation in family 1. The intensity of the exon 3 deletion bands was similar between families 1 and 2. However, it seemed that the clinical phenotype was a bit severe in family 2 compared to family 1 based on the level of attrition and multiple pulp exposures. 

Even though DGI-III was described as the most severe type of hereditary dentin defects and there are indeed additional defective features to those of DGI-II, there does not seem to be clear-cut, distinguishing boundaries between DGI-II and DGI-III [2,9]. The *DSPP* mutation causing DGI-III in the Brandywine isolate (c.49C>T, p.(Pro17Ser)) was identified [18], but the same mutation was identified in the other families with DGI-II [20,21]. Likewise, additional families with DGI-III features have been identified to be caused by different *DSPP* mutations [19,34]. Enlarged pulp chambers in young children have also been reported in several cases of DGI-II [20,24,35]. These variations may be attributable to genetic background and/or genetic modifiers. They may also be impacted by nongenetic factors. Masticatory force and dietary habits could influence the level of attrition and therefore, multiple pulp exposure as well. Inflammation and resultant periapical abscess occurring in exposed or near-exposed pulp tissue can also be influenced by nongenetic factors including oral hygiene care and dental caries development.

The synonymous change identified in family 3 was, in fact, a splicing mutation similar to the other two mutations. However, this silent mutation (c.135G>A, p.(Gln45=)) resulted in a much weaker exon 3 deletion band compared to the other two mutations in addition to a similar level of mutant transcript using the cryptic splicing donor site. Indeed, the clinical phenotype of family 3 was milder than those of families 2 and 3 and was diagnosed as DD-II. 

The translated mutant protein generated by the mutations in this study would be the same exon 3 deleted DSPP without the conserved IPV motif at the N-terminus after cleavage of the signal peptide, which is essential for efficient trafficking to be secreted via the Golgi apparatus [33]. The protein trafficking problem caused by the defective IPV domain was shown to result in ER retention of the mutant protein [34,36]. Retention of the mutant DSPP in the ER can cause one or both of the following effects. The mutant protein itself would act dominant negatively to cause damage to the odontoblasts and (pre-)ameloblasts. On the other hand, the mutant protein retained would interact with wild-type DSPP or other matrix proteins via the calcium-binding repeat domain and reduce the secretion of matrix proteins. Usually, ER overload or misfolded proteins cause ER stresses and cellular pathosis, even to cell death. However, interestingly, it seems that the conventional ER stress pathway markers do not change with mutant DSPPs [37]. Do the mutant DSPPs not actually cause ER pathosis? Alternatively, is there an unknown pathway yet to be identified? Further studies are necessary to characterize disease-causing mechanisms involving cellular damages.

In addition to cellular pathosis, secreted mutant DSPPs also need to be considered even if the amount is reduced because it has been shown that the DSP and DPP have different functional roles [38,39] and they are cleaved in the matrix after secretion [40,41]. Therefore, according to the type and location of the mutations, cleaved DSP and DPP may have different impacts in the developing matrix. For example, if the mutation is in the DSP domain, the cleaved DPP may retain normal function even though the amount may be reduced. Likewise, if the mutation is in the DPP domain, as in one of the frameshift mutations, the DSP domain will retain its normal function, but the DPP domain, having a different length of the hydrophobic chain of the mutant protein, may drastically alter the developing dentin and its mineralization.

## 5. Conclusions

We identified three novel *DSPP* mutations in two Turkish families and one Caucasian family with clinical phenotypes varying from DD-II to DGI-III. All three mutations including a silent mutation altered the pre-mRNA splicing machinery and, interestingly, the amounts of translated exon 3 deletion transcript correlated with the severity of dentin defects. This study is not only expanding the mutational spectrum of hereditary dentin defects caused by *DSPP* mutations, but also improving our understanding of the molecular pathogenesis of the disorder. Since the disease mechanism of hereditary dentin defects is complex, as reported in the literature and this study, further functional studies with full genomic fragments instead of minigene assay and animal diseases models are necessary.

## Data Availability

The data presented in this study are openly available in ClinVar (http://www.ncbi.nlm.nih.gov/clinvar (accessed on 18 May 2022)), Accession ID: SCV002104181, SCV002104182 and SCV002104183.

## Supplementary Materials

The following are available online at https://www.mdpi.com/article/10.3390/jpm12061002/s1, Table S1: Statistics for exome sequencing; Table S2: Mutagenesis primers; Figure S1: Clinical photos and panoramic radiographs of family 1; Figure S2: Clinical photos and panoramic radiographs of family 2; Figure S3: Clinical photos and panoramic radiographs of family 3; Figure S4: Mutation and sequencing chromatograms of family 1; Figure S5: Mutation and sequencing chromatograms of family 2; Figure S6: Mutation and sequencing chromatograms of family 3.
