# Supplementary material for: Translated Mutant DSPP mRNA Expression Level Impacts the Severity of Dentin Defects
Source: J Pers Med. 2022 Jun 19;12(6):1002. doi: 10.3390/jpm12061002 (PMC9225647; doi:10.3390/jpm12061002)
Supplement: Supplementary file 1 [file jpm-12-01002-s001.zip › jpm-1756997-supplementary.pdf]

Translated Mutant *DSPP* mRNA Expression Level Impacts the Severity of Dentin Defects

Youn Jung Kim[1], Yejin Lee[1], Hong Zhang[2], Figen Seymen[3], Mine Koruyucu[4], Sule Bayrak[5], Nuray Tuloglu[5], James P. Simmer[2], Jan C.-C. Hu[2], Jung-Wook Kim[1,6*]

[1]Department of Pediatric Dentistry & Dental Research Institute
School of Dentistry, Seoul National University, Seoul, Korea.
[2]Department of Biologic and Materials Sciences, School of Dentistry
University of Michigan, Ann Arbor, MI, USA.
[3]Department of Paediatric Dentistry, Faculty of Dentistry
Altinbas University, Istanbul, Turkey.
[4]Department of Paediatric Dentistry, Faculty of Dentistry
Istanbul University, Istanbul, Turkey.
[5]Private Practice, Eskisehir, Turkey.
[6]Department of Molecular Genetics & Dental Research Institute
School of Dentistry, Seoul National University, Seoul, Korea.

***Correspondence to:**

Jung-Wook Kim, D.D.S., M.S.D, Ph.D.
Department of Molecular Genetics
Department of Pediatric Dentistry & Dental Research Institute
School of Dentistry, Seoul National University
101 Daehak-ro, Jongno-gu, Seoul, 03080, Korea
Tel: +82-2-2072-2639, Fax: +82-2-744-3599
E-mail: pedoman@snu.ac.kr

Table S1. Statistics for exome sequencing.

Sample		Total reads	Mapping rate (%)	Median target coverage	Coverage of target region (%)	Fraction of target covered with at least:	
						20X	10X
Family 1	II:1	69,572,039	99.7	50	94.6	86.4	93.1
Family 3	I:2	155,617,078	99.9	122	96.4	94.7	95.9
Family 3	II:2	273,518,758	99.9	195	96.5	95.3	96.0

Table S2. Mutagenesis primers.

Primer name	Sequences
hDSPP_c.52-2del-F	5'-GTATTTTCTACTTGGCGGTTCCTCAAAGCAAACC-3'
hDSPP_c.52-2del-R	5'-GGTTTGCTTTGAGGAACCGCCAAGTAGAAAATAC-3'
hDSPP_c.135+1G>C-F	5'-ATGTGTCAGTACAGCTATAGGATGTAAT-3'
hDSPP_c.135+1G>C-R	5'-ATTACATCCTATAGCTGTACTGACACAT-3'
hDSPP_c.135G>A-F	5'-AATGTGTCAGTACAAGTATAGGATGTAAT-3'
hDSPP_c.135G>A-R	5'-ATTACATCCTATACTTGTACTGACACATT-3'

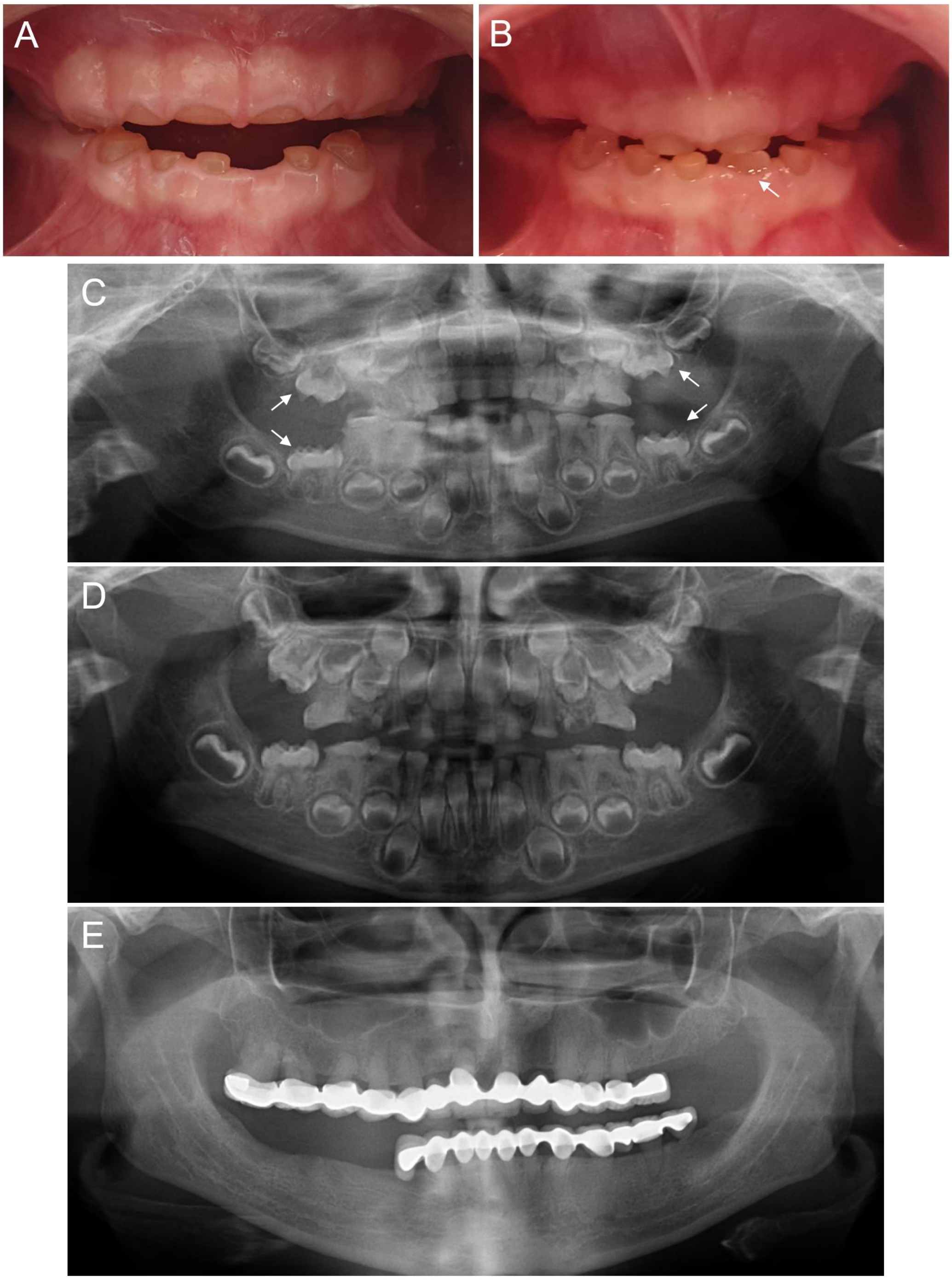

Figure S1. Clinical photos and panoramic radiographs of family 1. (**A**) Clinical photo of the proband in centric occlusion at age 5. Generalized severe attrition can be seen with anterior open bite. (**B**) Clinical photo of the sibling (III:2) of the proband in centric occlusion at age 5. Erupting mandibular left permanent central incisor (white arrow) exhibits amber–brown discoloration. (**C**) Panoramic radiograph of the proband at age 5 shows defective enamel coverage in the developing first molars (white arrows). Pulp chambers are obliterated mostly but not completely, especially in the deciduous canines. (**D**) Panoramic radiograph of the sibling (III:2) of the proband at age 5 shows similar findings to the panoramic radiograph of the proband at age 5. (**E**) Panoramic radiograph of the mother (II:4) of the proband at age 29 shows full mouth prosthodontics with multiple teeth absent due to extractions. Her remaining teeth have short and blunt roots with complete pulpal obliteration.

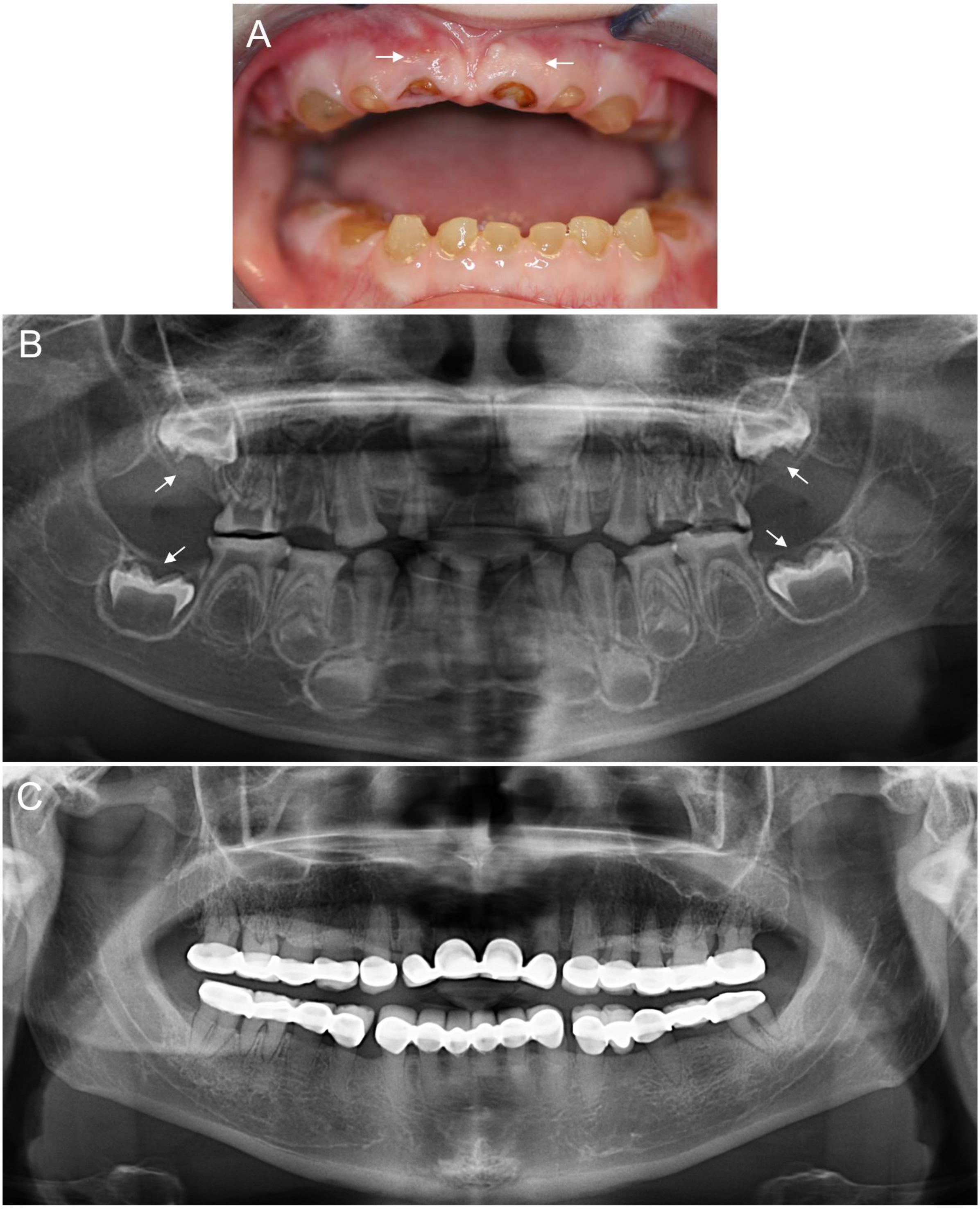

Figure S2. Clinical photos and panoramic radiographs of family 2. (**A**) Frontal clinical photo of the proband at age 4. Generalized severe attrition is evident. Pulp exposures and periapical abscesses can be seen in the deciduous maxillary central incisors (white arrows). (**B**) Panoramic radiograph of the proband at age 4 shows severe attrition, especially in the maxillary teeth. However, the pulp chambers are not obliterated. The enamel layer is abnormally shaped and its mineralization is reduced, especially in the first molars (white arrows). (**C**) Panoramic radiograph of the mother (I:2) of the proband at age 34 shows full mouth prosthodontics with several teeth extracted. Her remaining teeth show short and blunt roots with complete pulpal obliteration.

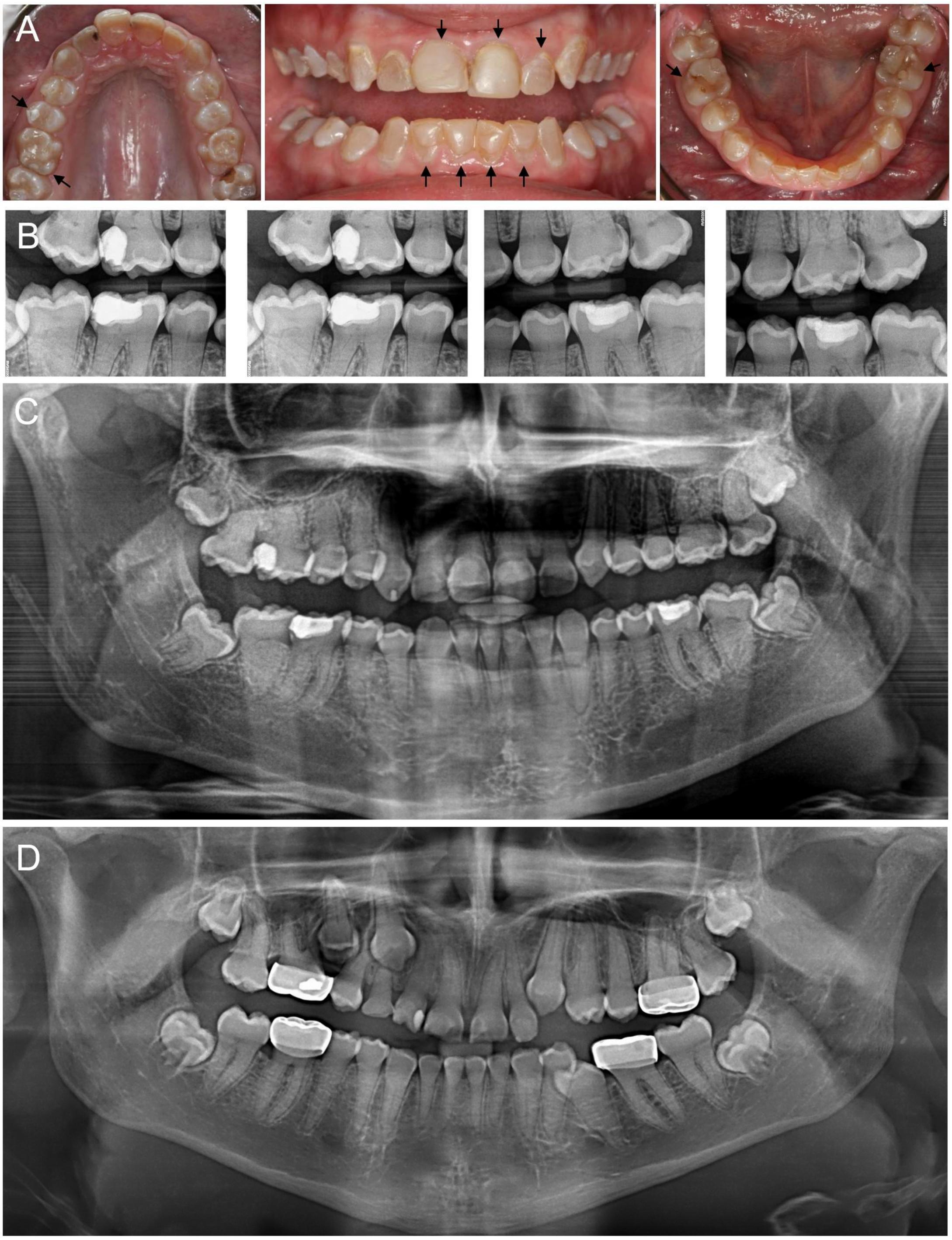

Figure S3. Clinical photos and panoramic radiographs of family 3. (**A**) Frontal and occlusal clinical photos of the sister (II:2) of the proband at age 17. Labial surfaces of several anterior teeth are covered with resin composite (black arrows). Several teeth including mandibular first molars are also restored (black arrows). Discoloration and attrition are mildly similar to that of the proband. (**B**) Bitewing radiographs of the sister (II:2) of the proband at age 17. The pulp chambers are more obliterated than that of the proband's. (**C**) Panoramic radiograph of the sister (II:2) of the proband at age 17 shows progressed pulpal obliterations in almost all teeth. (**D**) Panoramic radiograph of the brother (II:3) of the proband at age 15 shows remaining maxillary deciduous canines, but the pulp obliteration is almost complete in the crowns; the remaining pulp chambers can be seen in the roots. The thistle-tube-shaped remaining pulp chambers are not evident.

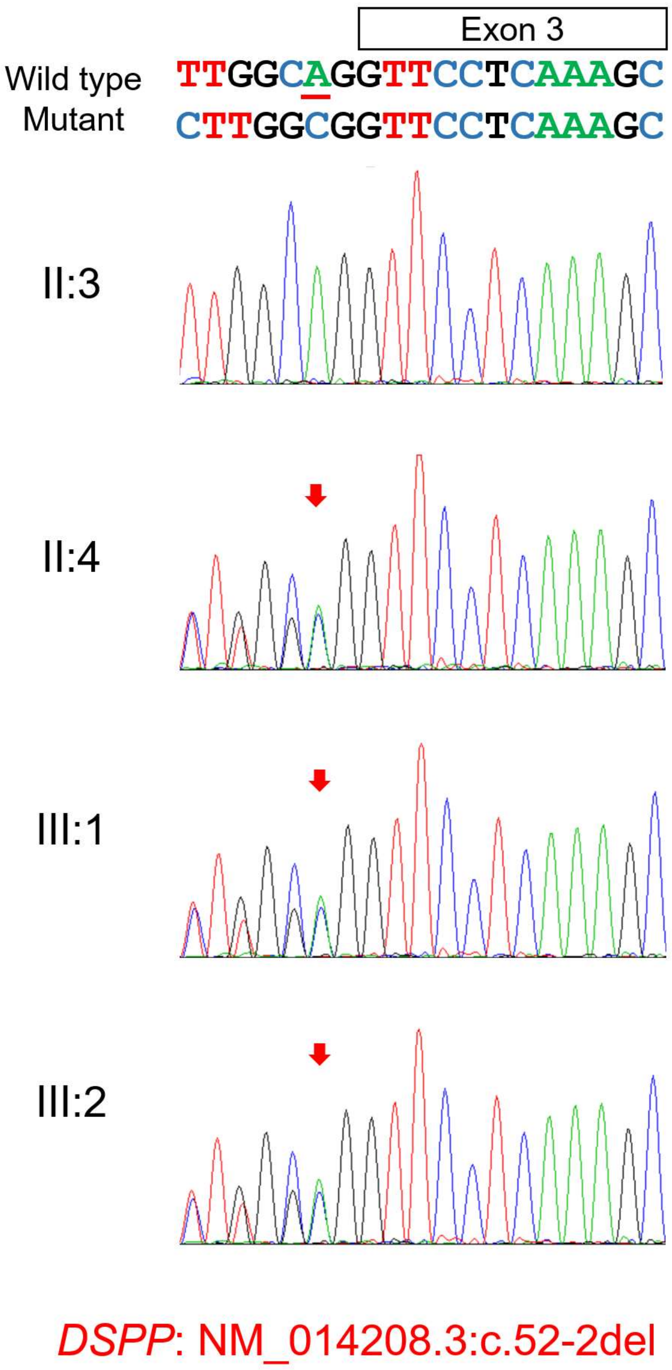

Figure S4. Mutation and sequencing chromatograms of family 1. Wild-type and mutant sequences are shown in the above chromatograms. Nucleotides belonging to exon 3 are shown by a box above the sequence. The deleted nucleotide is indicated by a red underline. Individual identification numbers are shown on the left side of the chromatograms. The location of the mutation is indicated with red arrows.

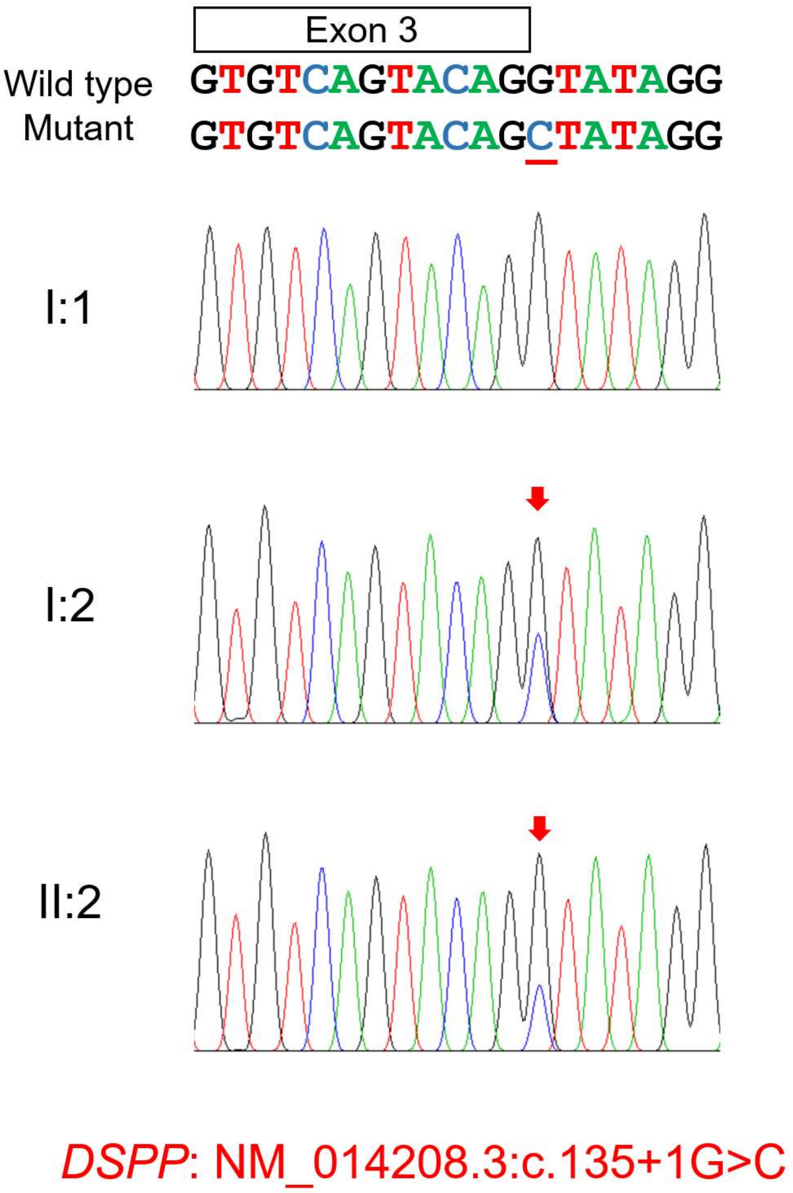

Figure S5. Mutation and sequencing chromatograms of family 2. Wild-type and mutant sequences are shown in the above chromatograms. Nucleotides belonging to exon 3 are shown by a box above the sequence. The mutated nucleotide is indicated by a red underline. Individual identification numbers are shown on the left side of the chromatograms. The location of the mutation is indicated with red arrows.

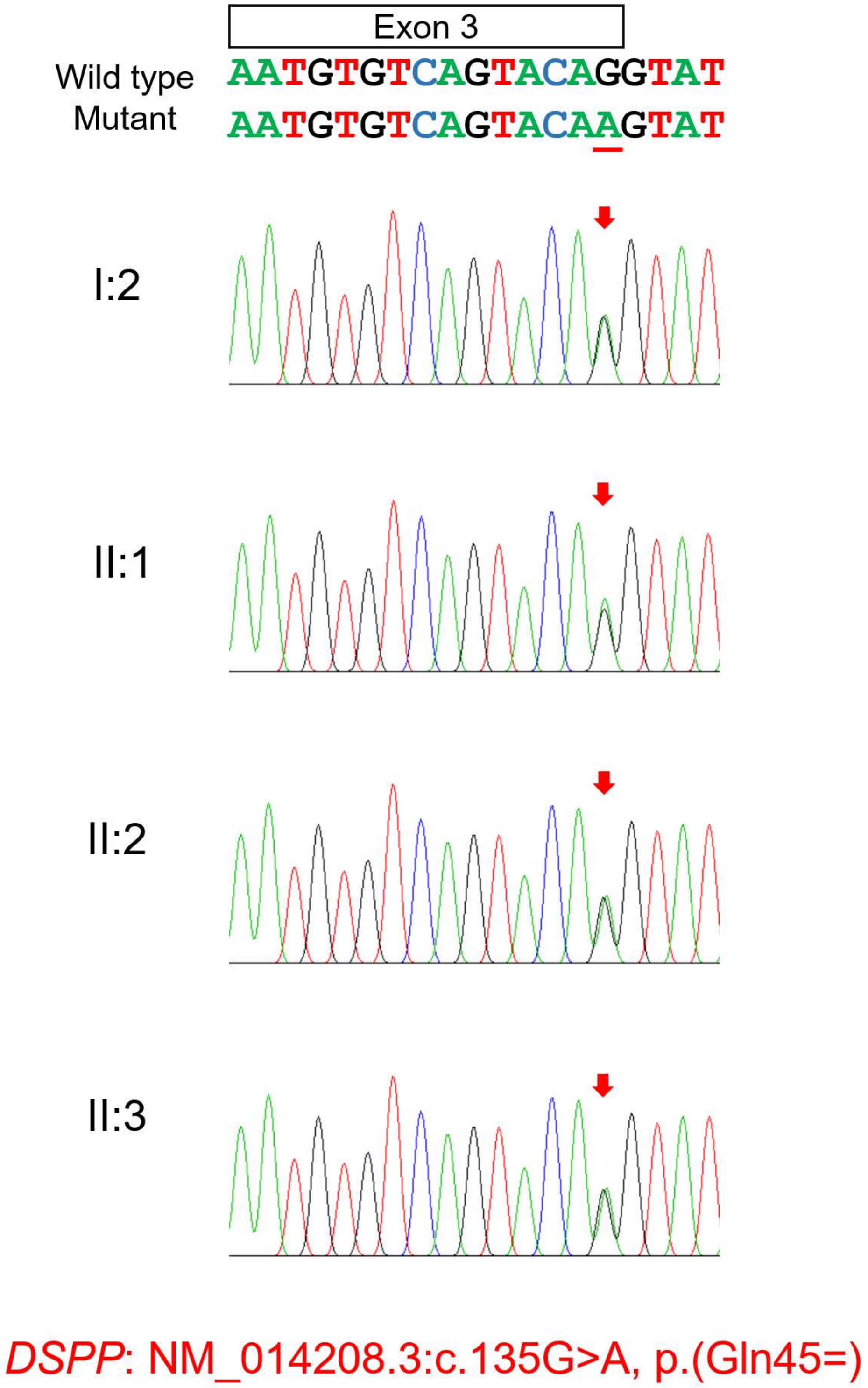

Figure S6. Mutation and sequencing chromatograms of family 3. Wild-type and mutant sequences are shown in the above chromatograms. Nucleotides belonging to exon 3 are shown by a box above the sequence. The mutated nucleotide is indicated by a red underline. Individual identification numbers are shown on the left side of the chromatograms. The location of the mutation is indicated with red arrows.
